# Clinical, genetics and in silico analysis of a novel *BSCL2* variant in a patient with CGL2 from Iranian Azeri Turkish ethnic group: expanding the genotypic spectrum through a comparative review

**DOI:** 10.1038/s41439-026-00350-6

**Published:** 2026-05-04

**Authors:** Hadi Bazmi, Neda Jabbarpour, Asma Alizadeh Asghari, Morteza Bonaydi, Akram Motamedi, Mohammad Barzegar

**Affiliations:** 1 Department of Animal Biology, Faculty of Natural Sciences, University of Tabriz, Tabriz, Iran; 2 Department of Pediatrics, Boali Hospital, Faculty of Medicine, Ardabil University of Medical Sciences, Ardabil, Iran; 3 Pediatric Health Research Center, Tabriz University of Medical Sciences, Tabriz, Iran

**Keywords:** Clinical genetics, Genomics

## Abstract

Congenital generalized lipodystrophy type 2 is a rare autosomal recessive disorder caused by mutation in the *BSCL2* gene. Here we report a novel variant (NM_001122955.4:c.828_835dup p.(Arg279ProfsTer21)) in an 18-year-old female with congenital generalized lipodystrophy type 2. The patient presented with severe lipoatrophy, muscular hypertrophy and insulin resistance. This frameshift variant introduces a premature stop codon, probably triggering nonsense-mediated decay. This finding expands the *BSCL2* mutational spectrum and highlights the importance of genetic analysis in consanguineous populations.

Lipodystrophy is a rare but clinically important disorder characterized by the complete or partial absence of adipose tissue in parts of the body^1^. Congenital generalized lipodystrophy type 2 (CGL2) is one of the most severe types of genetic lipodystrophy, characterized by generalized lipoatrophy from birth, which typically leads to a prominent muscular appearance, acromegaloid features (such as enlarged hands and feet and a triangular face) and phlebomegaly^2–5^. Metabolically, patients suffer from severe insulin resistance, early-onset diabetes mellitus, extreme hypertriglyceridemia and low serum leptin levels, often accompanied by acanthosis nigricans. Major organ involvement includes hepatosplenomegaly, hepatic steatosis and hypertrophic cardiomyopathy^2–5^. The seipin protein, which is encoded by this gene, is expressed in adipose tissue, brain and testicles and plays an important role in lipid droplet biogenesis and adipocyte differentiation (MIM: #606158)^6,7^. We present a case of CGL2 caused by a novel *BSCL2* mutation. Beyond documenting this new variant, our report enhances the understanding of CGL2’s genetic and phenotypic spectrum and underscores the value of genetic testing in patients with compatible clinical features.

The proband is an 18-year-old female from northwest Iran, born to consanguineous parents (first cousins). According to her mother, failure to thrive was evident from 2 months of age. A congenital hip dislocation (developmental dysplasia of the hip) was diagnosed and surgically corrected in infancy. Over time, she developed generalized lipoatrophy affecting the buttocks, breasts and buccal fat pads. This was accompanied by proximal limb wasting and marked muscular hypertrophy of the calves and forearms (Fig. 1).

**Fig. 1:**
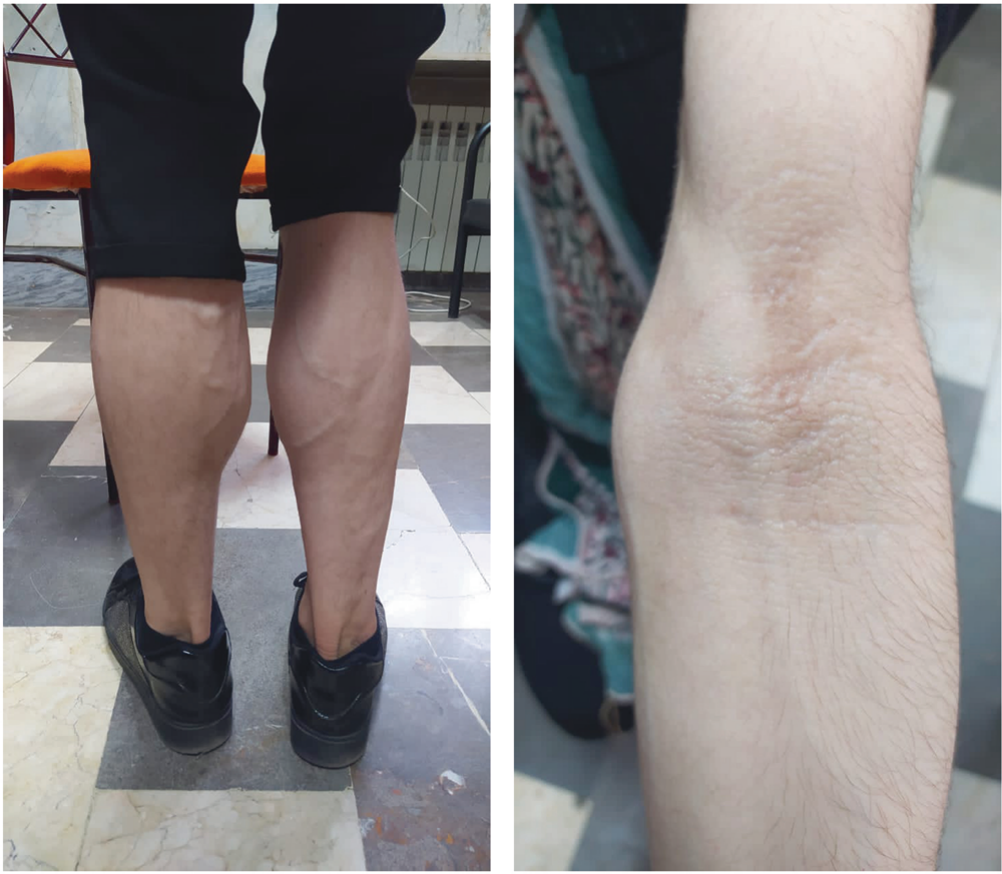
Clinical photograph demonstrating the characteristic findings of proximal limb wasting with concurrent muscular hypertrophy in the calves and forearms.

Dysmorphic features included a triangular face, macrognathia with prognathism, an acromegaloid body habitus, arachnodactyly and prominent superficial veins (phlebomegaly). Although initial symptoms suggested Russel–Silver syndrome, this diagnosis was reconsidered as her phenotype evolved.

Cutaneous manifestations consisted of coarse dry skin with eczematous changes, acanthosis nigricans, hypertrichosis, hyperhidrosis and multiple skin tags in flexural regions. Metabolically, she presented with severe insulin resistance, insulin-dependent type 2 diabetes mellitus (fasting blood sugar 255 mg/dl, HbA1c 9.06%), hypertriglyceridemia (triglyceride 773 mg/dl), low high-density lipoprotein (30 mg/dl) and hepatomegaly with elevated liver enzymes. She also had a history of acute pancreatitis requiring laparotomy at 17 years of age.

Motor developmental delay was observed; she began walking at 18 months. There was no evidence of intellectual disability. The family history was significant for multiple relatives with similar features who died in childhood from complications of severe recurrent pancreatitis and presumed sepsis, supporting an autosomal recessive inheritance pattern.

Whole-exome sequencing (WES) was carried out using the Agilent SureSelect Human All Exon V7 kit on an Illumina NovaSeq 6000 platform, and reads were aligned to the GRCh37/hg19 reference genome. The identified variant was validated in the proband and her parents by Sanger sequencing using specific primers (forward: 5′-TGAGGCGGGTAAGAGTGCTA-3′, reverse: 5′-TTGCCCAAGGTTCACTCCAG-3′). WES analysis identified a homozygous variant in the *BSCL2* gene at chr11:62459877_62459884. This alteration, c.828_835dupCTACCTCC, is located in exon 6 of transcript NM_001122955.4. It causes a frameshift, resulting in a premature termination codon (PTC) at p.Arg279Profs*21. While the specific protein and nucleotide changes vary across other *BSCL2* transcripts, they consistently lead to a PTC, suggesting a loss of protein function. To validate the WES finding, Sanger sequencing was performed using primers specific to this region, which confirmed the homozygous duplication in the proband. A graphical representation of the variant is provided in Fig. 2.

**Fig. 2:**
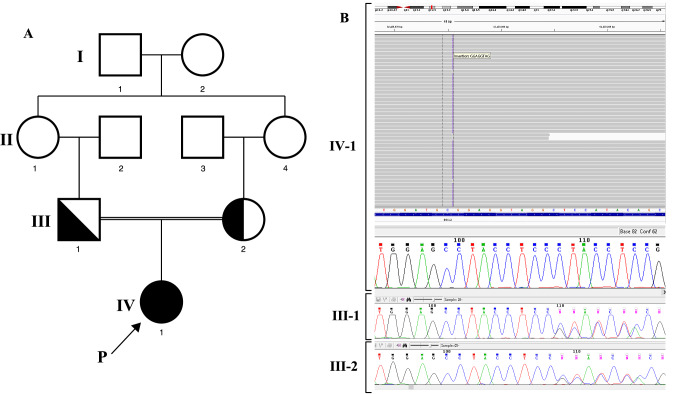
Genetic analysis of the identified novel mutation. **A** Pedigree showing consanguinity and autosomal recessive inheritance. Both parents are carrier for the mutation and the proband is homozygous for the mutation. **B** Identification of a homozygous *BSCL2* mutation in the proband. **Top panel**: Integrative Genomics Viewer (IGV) screenshot of the WES BAM file from the proband, showing the duplicated region (c.828_835dupCTACCTCC) in the *BSCL2* gene. **Bottom panel**: Sanger sequencing chromatograms, confirming the homozygous presence of the mutation in the proband (top). The parents are heterozygous carriers of the same mutation (bottom), confirming autosomal recessive inheritance.

The variant was absent from the ClinVar database^8^, indicating it is novel. This absence was corroborated by the Franklin and Varsome interpretation platforms, which reported no occurrences in population databases such as gnomAD (v4.1)^9^, ExAC^10^ or the 1000 Genomes Project^11^, resulting in an allele frequency of zero. Furthermore, the variant was not identified in a local cohort of about 500 individuals from northwest Iran who underwent WES.

Genetic analysis of our patient revealed a novel eight-nucleotide duplication in *BSCL2*, resulting in a frameshift and a PTC. Consistent with the consanguineous family history, the variant was homozygous, confirming an autosomal recessive inheritance pattern. According to American College of Medical Genetics and Genomics guidelines^12^, this variant meets the criteria for PVS1 (predicted loss of function in a gene where this is a known mechanism of disease) and PM2 (absent from population databases), supporting its classification as likely pathogenic^12^. Mutations in *BSCL2*, including missense, nonsense, frameshift and splice-site variants, have all been reported to cause seipin dysfunction^13,14^. Our finding aligns with this diverse mutational landscape and reinforces the link between loss-of-function mutations and the severe CGL2 phenotype.

Our patient exhibited all typical CGL2 features, with the exception of intellectual disability and precocious puberty. Notably, the patient also presented with pancreatitis, a rare complication in CGL2^5,15^. This underscores the disease’s clinical variability and highlights the importance of considering atypical manifestations during diagnosis.

Early detection of autosomal recessive diseases like CGL2 is crucial in consanguineous populations. Genetic counseling enables informed reproductive decisions and prevention of new cases, although diagnosis often occurs only after severe complications arise^16^. Phenotypic variation can further delay diagnosis, a problem exacerbated by the social burden of the disease and limited access to specialized care^17^.

Our findings identify a novel *BSCL2* variant linked to a classic CGL2 phenotype. This variant is predicted to cause a loss of seipin function via nonsense-mediated decay triggered by a premature stop codon. Our study highlights the critical role of early genetic testing in managing CGL2 and expands the mutational spectrum of *BSCL2*, while also pointing to the necessity of functional validation to definitively confirm the disease mechanism.

## HGV Database

The relevant data from this Data Report are hosted at the Human Genome Variation Database via Figshare at 10.6084/m9.figshare.hgv.3654 (ref. ^18^).

## Data Availability

The datasets generated or analyzed during the current study are available from the corresponding author on reasonable request.
